# How Often Should Microbial Contamination Be Detected in Aircraft Fuel Systems? An Experimental Test of Aluminum Alloy Corrosion Induced by Sulfate-Reducing Bacteria

**DOI:** 10.3390/ma17143523

**Published:** 2024-07-16

**Authors:** Bochao Lu, Yimeng Zhang, Ding Guo, Yan Li, Ruiyong Zhang, Ning Cui, Jizhou Duan

**Affiliations:** 1 Key Laboratory of Advanced Marine Materials, Key Laboratory of Marine Environmental Corrosion and Biofouling, Institute of Oceanology, Chinese Academy of Sciences, Qingdao 266071, China; lubochao999@163.com (B.L.); 17860718330@163.com (D.G.); ruiyong.zhang@qdio.ac.cn (R.Z.); duanjz@qdio.ac.cn (J.D.); 2 School of Mechanical and Automotive Engineering, Qingdao University of Technology, Qingdao 266520, China; 3 Qingdao Campus of Naval Aviation University, Qingdao 266041, China

**Keywords:** *Desulfovibrio*, biocorrosion, aluminum alloy, fuel oil, corrosive environments

## Abstract

Microbial contamination in aircraft fuel-containing systems poses significant threats to flight safety and operational integrity as a result of microbiologically influenced corrosion (MIC). Regular monitoring for microbial contamination in these fuel systems is essential for mitigating MIC risks. However, the frequency of monitoring remains a challenge due to the complex environmental conditions encountered in fuel systems. To investigate the impact of environmental variables such as water content, oxygen levels, and temperature on the MIC of aluminum alloy in aircraft fuel systems, orthogonal experiments with various combinations of these variables were conducted in the presence of sulfate-reducing bacteria. Among these variables, water content in the fuel oil demonstrated the most substantial influence on the corrosion rate of aluminum alloys, surpassing the effects of oxygen and temperature. Notably, the corrosion rate of aluminum alloys was the highest in an environment characterized by a 1:1 water/oil ratio, 0% oxygen, and a temperature of 35 °C. Within this challenging environment, conducive to accelerated corrosion, changes in the corrosion behavior of aluminum alloys over time were analyzed to identify the time point at which MIC intensified. Observations revealed a marked increase in the depth and width of corrosion pits, as well as in the corrosion weight-loss rate, starting from the 7th day. These findings offer valuable insights for determining the optimal frequency of microbial contamination detection in aircraft fuel systems.

## 1. Introduction

Corrosion has emerged as a significant impediment to economic development, environmental preservation, and safety, resulting in a substantial 3.34% loss in China’s Gross Domestic Product (GDP) [1]. Metallic materials in aircraft structures are susceptible to corrosion, accounting for 23.6% of the total maintenance costs of the total sustainment costs for US Air Force aviation and missiles in the fiscal year of 2018 [2]. Among the wide range of corrosion forms, microbiologically influenced corrosion (MIC) is metal corrosion associated with the action of microorganisms present in the corrosion system. It is one of the significant contributors to the corrosion of man-made infrastructure and equipment in natural environments, especially in marine environments with high humidity, temperature, and salinity, as well as other harsh and extreme conditions [3]. Estimates suggest that losses attributable to MIC could constitute as much as 20% of total corrosion-related losses, with corrosion rates reaching up to 4 mm per year on metal materials [4].

Microbial contamination in maritime flight equipment, including seaplanes and carrier-based aircraft, particularly in oil-containing systems such as fuel pumps and fuel tanks of the aircraft, leads to serious consequences ranging from filter and injector fouling to engine malfunctions and accelerated equipment corrosion [5]. The microbial tests to monitor the microbial contamination in fuel and fuel systems were only commenced in 2002 following safety incidents attributed to MIC [6]. In such fuel-containing systems, hydrocarbons with C10–C18 carbon chains in fuel were shown to be degraded and serve as the carbon sources to support microbial growth. Additionally, nitrogen and inorganic salts from both fuel and non-metallic materials, such as nitrile rubber and polyurethane foam in the fuel tank, are also utilized for microbial growth and multiplication [7]. The fuel tank of an aircraft will inevitably contain water that is condensed from the fuel itself and the outside air during the process of transportation, daily use, and maintenance of the aircraft, supporting microbial growth [8]. The concentration of oxygen in the headspace of fuel systems can also significantly impact microbiologically influenced corrosion MIC. Furthermore, aircraft experience fluctuating temperatures throughout their flights. When conditions such as temperature, water presence, oxygen levels, nutrients, and other factors align to support microbial growth, a rapid proliferation of microorganisms occurs, leading to corrosion in the fuel tank and escalating maintenance and repair expenses [9,10,11]. Therefore, the assessment of microbial contamination in fuel-containing systems must consider the influence of the surrounding environment, including varying humidity, oxygen levels, and temperature, on microbial growth [12].

The contaminative microorganisms in aircraft fuel and fuel systems mainly include fungi and bacteria. Filamentous fungi with hydrocarbon-degrading ability, including *Aspergillus*, *Cladosporium*, and *Penicillium*, can be a major problem that causes corrosion by producing a variety of acids. In addition, facultative bacteria, such as *Bacillus*, and anaerobes, such as sulfate-reducing bacteria (SRB), are possible threats [13]. Among diverse contamination-causing microorganisms in maritime aircraft, SRB, with a sulfate-respiration metabolism, are the most threatened microbial populations to the corrosion of aviation fuel systems [14,15,16,17]. The corrosion mechanism of SRB has been studied extensively, including currently proposed chemical-MIC through an excreted chemical agent and electrical-MIC through direct electron uptake [18,19,20,21,22].

Aluminum alloys are widely used in aircraft as the skin, wing beam, partition frame, and integral fuel tank panel because of their good performance in terms of high corrosion resistance, high strength, and low density [23]. The dense oxide passivation film (mainly Al_2_O_3_) is formed on the surface of aluminum alloys to resist corrosion. Research on microbiologically influenced corrosion (MIC) in aviation fuel systems has predominantly focused on studies influenced by sulfate-reducing bacteria (SRB). The key finding from these studies suggests that SRB accelerate aluminum corrosion by disrupting the protective passivation film through the action of biogenic sulfide [24,25,26,27,28], while some research pointed out that the biofilms formed by SRB can also reduce corrosion by protecting the aluminum alloy from acidic corrosive substances [29]. The corrosion behavior of SRB is likely influenced by the surrounding environments within aviation fuel systems, including factors like water content, oxygen levels, and temperature [30], whereas comprehensive studies in this area are currently lacking.

To reduce the threat of microbial corrosion of aluminum alloy-made materials in the fuel systems of aircraft, it is necessary to detect and monitor microbial contamination of fuel periodically. However, a better understanding of the risk of microbial corrosion in the aircraft fuel system, as well as the growth features and corrosion behavior of corrosion-causing microorganisms under different environmental factors, is still lacking. There are no systematic and uniform detection standards for microbial detection in marine aircraft, especially how often the microbial contamination should be monitored.

To select the environment in which the MIC of aluminum alloys is accelerated the most, the orthogonal experiment enables a more effective way to identify the main factors influencing experimental results and facilitate comparison of the impact of different factors. The advantages of orthogonal experiments include the following: (1) effectively identifying the main factors influencing experimental results, reducing the consideration of complex factors and the number of experiments required, and (2) systematically studying the effects of multiple factors on experimental outcomes, facilitating comparison of the impact of different factors. As the multi-environmental factors were considered in the present study, the orthogonal experiment is an effective way to access MIC in these complex environments. The orthogonal experimental design here combined three environmental factors (water content, oxygen content, and temperature) with various gradient concentrations separated into nine different groups. The influence of environmental factors on microorganisms was clarified, and the combination of environmental factors with the greatest impact on MIC was screened out through this orthogonal experiment. Secondly, under such a combination of environmental factors that highly accelerate corrosion, a 14-day time immersion experiment was carried out to study the effects of a corrosion-causing SRB strain on aluminum alloy corrosion at different growth stages. Subsequently, under the identified combination of environmental factors that greatly accelerated corrosion, a 14-day immersion experiment was conducted to investigate the effects of a corrosion-causing sulfate-reducing bacteria (SRB) strain on aluminum alloy corrosion at different growth stages. It was observed that corrosion of the aluminum alloy accelerated notably from the 7th day, coinciding with a significant deterioration in fuel quality and heightened activity of SRB on the alloy surfaces. These findings offer valuable theoretical insights for establishing the detection cycle of contaminated microorganisms in maritime aircraft fuel systems.

## 2. Materials and Methods

### 2.1. Materials and Microbes

A commonly used aluminum alloy in aircraft manufacturing, 7B04 aluminum alloy, was chosen as the representative material in the present study. The chemical composition of 7B04 was as follows (wt %): Al 91.2, Si 0.10, Fe 0.05, Cu 1.40, Mn 0.2, Mg 1.80, Cr 0.10, Ni 0.10, Zn 5.0, and Ti 0.05. Firstly, the aluminum alloy plates were cut into 10 × 10 × 3 mm^3^ square coupons using a wire cutting machine and polished to 2000 grit with SiC papers step by step. Secondly, the coupons were washed with anhydrous ethanol and sonicated for 20 min at room temperature to remove the impurities attached to the surface. Finally, the coupons were dried in N_2_ atmosphere and placed in an anaerobic glove box. The electrochemical working electrode has a working area of 1 cm^2^, and the rest of the electrode is sealed with polytetrafluoroethylene. All coupons were placed under UV light for 40 min and then transferred to modified PGC medium. The UV lamp is a 750 W BBS-SDC model, and the distance between the UV lamp and the aluminum alloy coupon is 500 mm.

A marine sulfate-reducing bacteria, *D. bizertensis*, which was isolated from the corroded metals, was cultivated using a modified Postgate’s Medium C [31,32]. The modified Postgate’s Medium C contained 0.5 g KH_2_PO_4_, 1 g NH_4_Cl, 0.06 g CaCl_2_·6H_2_O, 0.06 g MgSO_4_·7H_2_O, 6 mL 70% sodium lactate, 1 g yeast extract, and 0.3 g sodium citrate in 1 L aged seawater from a Qingdao offshore area. Prior to testing, the medium pH was adjusted to 7.2 ± 0.1. L-cysteine was added to remove residual oxygen from the medium after N_2_ was passed through the medium for 30 min. The rubber stopper, culture medium, aeration needle, and cotton are sterilized in an autoclave at 121 °C for 20 min.

### 2.2. Orthogonal Experiments of Various Environmental Factors

In practical cases, the contaminated microbes in aircraft systems usually grow in various and complicated environments with diverse humidity, oxygen, and temperature levels. To determine the extent of environmental factors that affected MIC process, a statistical orthogonal experiment was set up. Various concentration gradients of these three environmental factors were combined randomly into 9 groups (Table 1). Each group was performed in triplicate. A volume of 1 mL homogenized *Desulfovibrio*-containing medium was inoculated into the anaerobic bottles that were filled with liquid medium composed of different water/oil ratios and with headspace composed of different oxygen/nitrogen ratios. The bacteria number of the *Desulfovibrio*-containing medium inoculated in each bottle was ~10^4^ cells/mL based on q-PCR tests. The 120 mL anaerobic flask used for the immersion experiment contained 50 mL of medium and was inoculated with 0.5 mL of SRB seeds; the 500 mL wide-mouth flask with stopper used for the electrochemical experiment contained 450 mL of medium and was inoculated with 4.5 mL of SRB seeds. The aluminum alloy coupons were put into these bottles, which were put into incubators with different temperatures, as shown in Table 1. The corrosion rate of these coupons, as shown by pit depth and average weight loss tests under the 9 conditions, was tested after 14 days of incubation, as described in the following part.

### 2.3. Corrosion Behavior of Aluminum Alloy Influenced by D. bizertensis under Harshest Conditions

After choosing the most corrosive conditions in which the aluminum alloy had the highest corrosion rate, corrosion behavior tests over time were performed to determine the time point at which the corrosion rate obviously increased under the influence of *D. bizertensis.* The tests of electrochemical behavior, surface morphology, mineralogy, and cell growth were performed as described below.

The OD values were observed using a spectrophotometer with an absorbance of 600 nm to characterize the planktonic cell count. The initial inoculate concentration of bacterial cells was 10^8^ cells/mL. Sessile cells were rinsed using PBS to observe the counts (deionized water 1 L, KH_2_PO_4_ 0.27 g, Na_2_HPO_4_ 1.42 g, KCl 0.2 g, NaCl 8 g). Observed and counted in a light microscope at 400× magnification using a blood cell counter.

### 2.4. Biofilm and Corrosion Product Film Characterizations

The coupons were removed from the medium and immersed in glutaraldehyde solution for 2 h to immobilize the bacteria. Stage-by-stage dehydration using anhydrous ethanol (10%, 20%, 30%, 40%, 50%, 60%, 70%, 80%, 90%, and 100% (*v*/*v*)), followed by blow-drying of the coupons under N_2_. Scanning electron microscopy (SEM) (Zeiss, Oberkochen, Germany) was used to observe the corrosion product films on aluminum alloy surfaces.

The distribution of live/dead bacteria on the surface of the coupon was observed using fluorescent microscope (FM) (Olympus, Tokyo, Japan) after the following steps. Firstly, rinse the surface of the specimen with PBS to remove the planktonic bacteria on the surface. Secondly, use a blotting paper to absorb the PBS solution on the surface. Finally, drop the stain evenly onto the surface of the specimen. Staining was carried out under dark conditions for 20 min for fluorescence observation.

X-ray diffraction (XRD) (Rigaku, Akishima-shi, Japan) and energy-dispersive spectroscopy (EDS) (INCAx, Oxford, UK) are used to research the chemical composition of corrosion product films on alloy coupons after collecting corrosion products as the following steps: open the anaerobic vial in a medical clean bench and remove the aluminum alloy coupons using forceps, then rinse the specimen in deionized water followed by anhydrous ethanol. Finally, the specimens were blown dry with N_2_ and sealed into 5 mL centrifuge tubes. MDI Jade software (version 6.0) was utilized to analyze data from XRD characterization.

### 2.5. Weight Loss and Pit Morphology

The weight loss of coupons is calculated by Equation (1):

(1) $$v_{corr}=\frac{m_{0}-m_{1}}{A}$$

The weight loss rate of coupons is calculated by Equation (2) [33]:

(2) $$v_{corr}=\frac{(m_{0}-m_{1})\times K}{A\times t\times \rho }$$

The pits on the surface of the coupons were observed using confocal laser scanning microscope (CLSM). Aluminum alloy coupons removed from the medium were rinsed with anhydrous ethanol and blown dry with N_2_. The coupons were immersed in HNO_3_ solution for 5 min to remove corrosion products firmly adhered to the surface based on GB/T 16545-2015 [34]. The Gaussian fit of the collected pitting data was performed.

### 2.6. Electrochemical Measurements

A series of electrochemical measurements of aluminum alloys in the culture medium were performed using an Gamry electrochemical workstation (Gamry, PA, USA). Electrochemical testing uses a three-electrode system: aluminum alloy as the working electrode, platinum electrode as the counter electrode, and saturated calomel electrode as the reference electrode. The 4.5 mL of *D. bizertensis* was inserted into 450 mL of medium. The electrochemical impedance spectroscopy (EIS) has a frequency range of 10^5^ Hz to 10^−2^ Hz with an amplitude of 10 mV. Potentiodynamic polarization ranged from −0.5 V to +1 V with respect to the open circuit potential (OCP), with a scan rate of 0.000167 V/s.

## 3. Results

### 3.1. Statistical Orthogonal Experiments under Various Environmental Conditions

**Figure 1:**
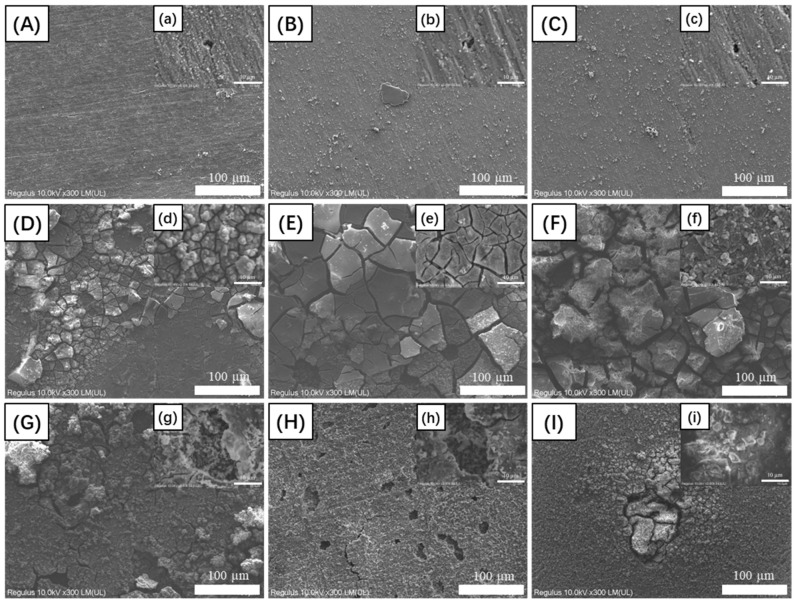
SEM images of the corrosion products on aluminum alloy coupons after the 14 d incubation with different environmental conditions: (**A**,**a**) for Group 1; (**B**,**b**) for Group 2; (**C**,**c**) for Group 3; (**D**,**d**) for Group 4; (**E**,**e**) for Group 5; (**F**,**f**) for Group 6; (**G**,**g**) for Group 7; (**H**,**h**) for Group 8; (**I**,**i**) for Group 9 in the fuel-containing media.

Figure 1 shows the SEM images of corrosion products covered by aluminum alloy coupons after the 14 d incubation in the fuel-containing media with different environmental conditions. It showed that the bacteria in fuel oil grew slowly and accumulated little corrosion products under the environmental conditions of Group 1, Group 2, and Group 3. However, the bacteria in fuel oil grew rapidly, and the thick corrosion product layer appeared on the aluminum alloy substrate after the 14 d incubation in Group 4, Group 5, and Group 6.

**Figure 2:**
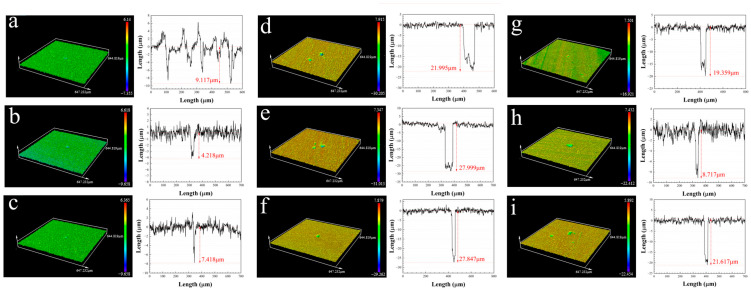
The CLSM images of coupons after the 14 d incubation with different environmental conditions: (**a**) for Group 1; (**b**) for Group 2; (**c**) for Group 3; (**d**) for Group 4; (**e**) for Group 5; (**f**) for Group 6; (**g**) for Group 7; (**h**) for Group 8; (**i**) for Group 9 in the fuel-containing media.

The depth and width of pits in the fuel-containing media after the 14 d incubation with different environmental conditions are shown in Figure 2. The pit depth profiles showed that the deepest pits of aluminum alloy coupons in Group 6 (water/fuel ratio of 1:1, obligately anaerobic (0%) and 35 °C) were 27.8 μm, which was higher than that of other groups. This indicates that *D. bizertensis* induced the most severe pitting corrosion under the environmental conditions of Group 6.

**Figure 3:**
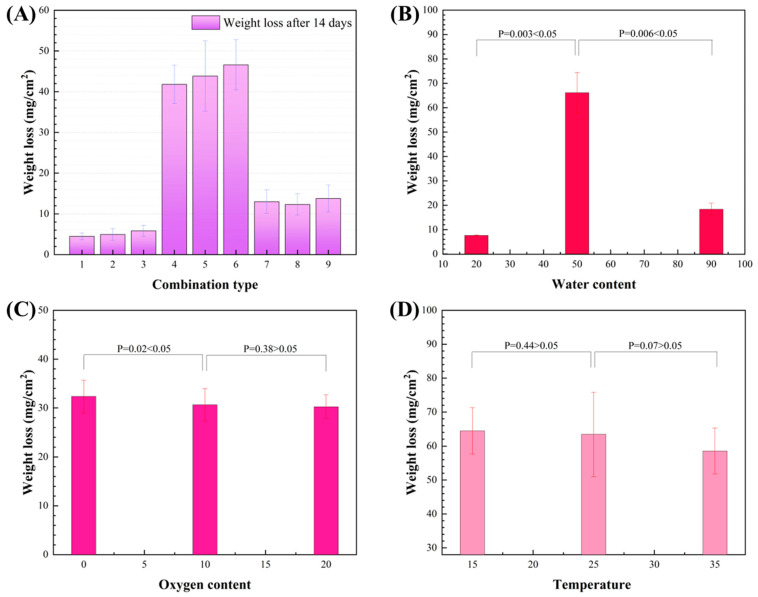
Weight loss of aluminum alloy coupons in the fuel-containing media with different environmental conditions after the 14 d incubation (**A**), and the influence of water content (**B**), oxygen content (**C**), and temperature (**D**) on weight loss of aluminum alloy coupons.

Figure 3A shows the weight loss of aluminum alloy coupons with different environmental conditions after the 14 d incubation. The corresponding corrosion rates of Group 1, Group 2, and Group 3 were 4.4 ± 0.8 mg/cm^2^, 4.9 ± 1.4 mg/cm^2^, and 5.8 ± 1.3 mg/cm^2^, respectively. These three groups had no obvious difference. The corrosion rate for Group 6 was 46.6 ± 6.1 mg/cm^2^, which was 10.5-fold, 9.5-fold, and 8.1-fold higher than that of Group 1, Group 2, and Group 3, respectively. The results of the weight loss test are in accordance with the pit corrosion.

The extent of the effect of three individual environmental factors on corrosion was also analyzed (Figure 3B–D). The corrosion weight loss increased significantly along with increasing water concentration when the water concentration was below 50% but decreased significantly along with increasing water concentration when it was below 50% (Figure 3B). When the oxygen content was in the range of 0–10%, the corrosion decreased significantly with the increase in the oxygen content. When the oxygen content was 10–20%, the corrosion did not decrease significantly. The effect of temperature on the whole corrosion process is not significant. Furthermore, according to the orthogonal experiment, we used the R-value to express the degree of influence of three environmental factors on corrosion. A larger R-value means that environmental factors have a greater influence on corrosion. The corrosion index indicated by the R-value of the aluminum alloy coupons were 44.8, 2.63, and 6.8, respectively, showing the same result. Thus, water content in fuel oil has the greatest impact on the MIC of aluminum alloy among the three environmental factors.

In conclusion, *D. bizertensis* influenced the corrosion of aluminum alloy the most under Group 6 conditions, namely with a water/fuel ratio of 1:1, obligately anaerobic (0% oxygen), and a temperature of 35 °C. Water concentration influenced the MIC of aluminum alloy the most among the three environmental factors in fuel-containing systems. The following experiments were performed in the Group 6 environment.

### 3.2. Bacterial Growth and Biofilm Characterization

**Figure 4:**
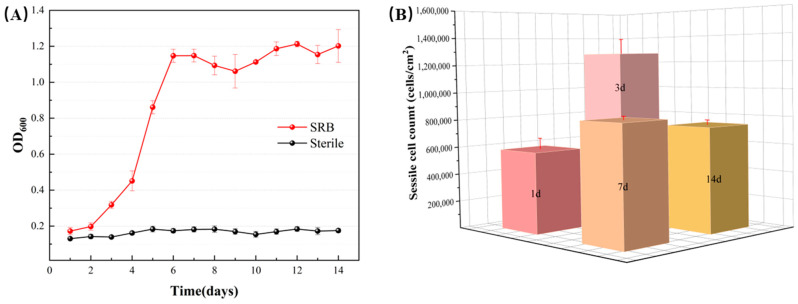
*D. bizertensis* planktonic cell count (**A**) during the 14 d incubation and sessile cell count (**B**) on aluminum alloy coupon surfaces with different immersion times.

The growth curves of sessile and planktonic *D. bizertensis* during the 14 d incubation on aluminum alloy coupons in the *D. bizertensis* media with different immersion times are shown in Figure 4. The growth curves of *D. bizertensis* can be divided into three phases: linear, stable, and decay. *D. bizertensis* showed an exponential growth stage in the first 6 days, when cells grew rapidly, and the planktonic cell counts reached about 10^8^ cells/mL on the 7th day. From the 7th day, cell growth slows down and enters a stagnant period.

**Figure 5:**
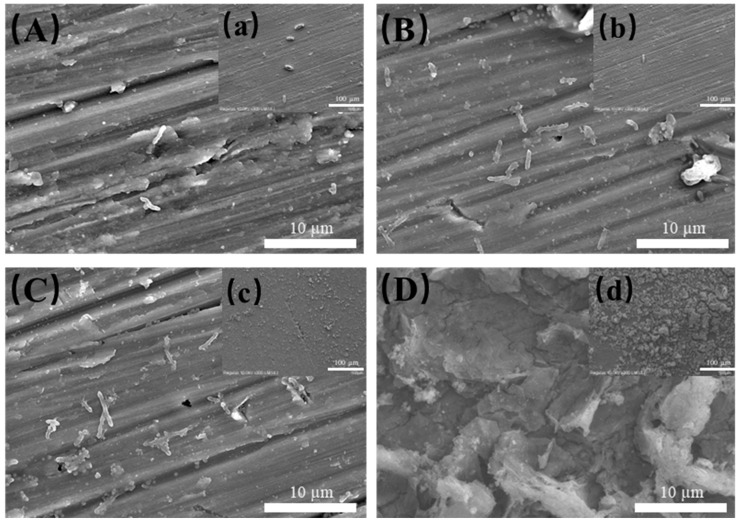
SEM images of the *D. bizertensis* biofilm and the corrosion products on aluminum alloy coupons during the 14 d incubation with different immersion times: (**A**) and (**a**) for 1 d; (**B**) and (**b**) for 3 d; (**C**) and (**c**) for 7 d; (**D**) and (**d**) for 14 d in the *D. bizertensis* media.

Figure 5 shows the SEM images of *D. bizertensis* and corrosion products on aluminum alloy coupons during the 14 d incubation in the *D. bizertensis* media with different immersion times. It showed that more and more *D. bizertensis* cells attached to surfaces over time and attended to form biofilms after 14 days.

**Figure 6:**
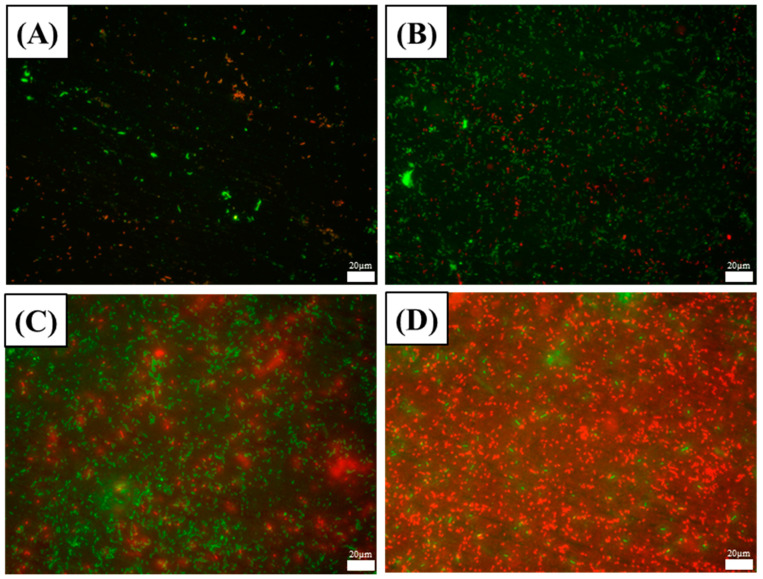
FM images of *D. bizertensis* biofilm on aluminum alloy coupons during the 14 d incubation: (**A**) for 1 d; (**B**) for 3 d; (**C**) for 7 d; (**D**) for 14 d in the *D. bizertensis* media.

To indicate the activity of the attached *D. bizertensis,* the live and dead cells on the surfaces of aluminum alloy coupons were stained using multiple fluorescence staining (Figure 6). Red and green areas denote dead and live *D. bizertensis* cells, respectively. Live *D. bizertensis* cells increased over time from the first day to the seventh day (Figure 6A–C). After the seventh day, the total cells reached a high number, while dead cells also began to increase over time (Figure 6D). This indicates high activities of attached cells from the 7th day, consistent with the results of sessile cell counts (Figure 4B) and biofilm observations (Figure 5).

### 3.3. Composition of Corrosion Products

**Figure 7:**
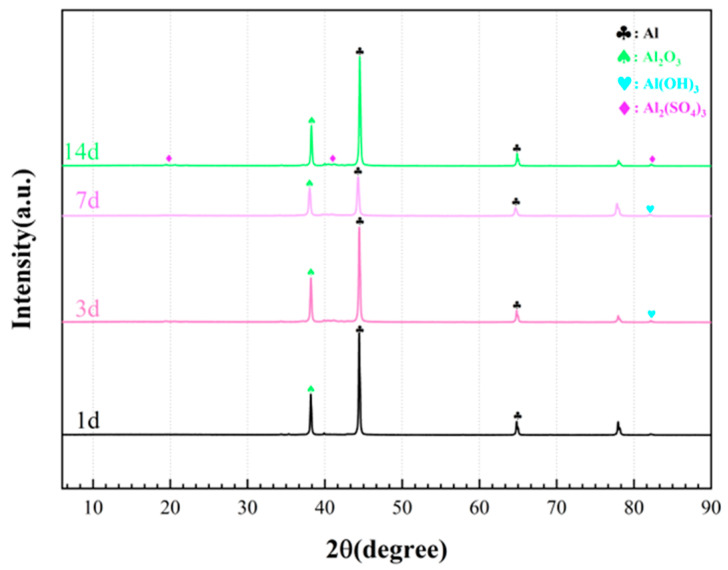
XRD pattern of the corrosion products on aluminum alloy coupons during the 14 d incubation in the *D. bizertensis* media with different immersion times.

Figure 7 illustrates the XRD patterns of the corrosion products on aluminum alloy coupons during the 14 d incubation in the *D. bizertensis* media. The characteristic peaks of Al_2_O_3_ and Al can be observed in the pre-corrosion immersion period. With increasing immersion time, the characteristic peaks of Al_2_(SO_4_)_3_ can be clearly observed.

### 3.4. The Pitting Corrosion

**Figure 8:**
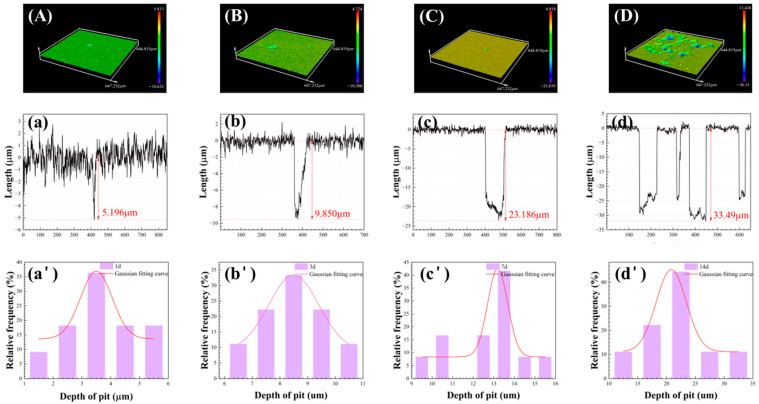
The CLSM images (**A**–**D**), the maximum depth of corrosion pits (**a**–**d**) and the statistical analysis of pits (**a’**–**d’**) during the 14 d incubation: (**A**) and (**a**,**a’**) for 1 d; (**B**) and (**b**,**b’**) for 3 d; (**C**) and (**c**,**c’**) for 7 d; (**D**) and (**d**,**d’**) for 14 d in the *D. bizertensis* media.

The pit morphology of corroded coupons during the 14 d incubation in the *D. bizertensis* media was observed (Figure 8). The number, diameter, and depth of the pits induced by *D. bizertensis* increased remarkably over time. The maximum pit depth was 5.1 µm, 9.8 µm, 23.1 µm, and 33.4 µm detected at 1 d, 3 d, 7 d, and 14 d, respectively.

### 3.5. Weight Loss

**Figure 9:**
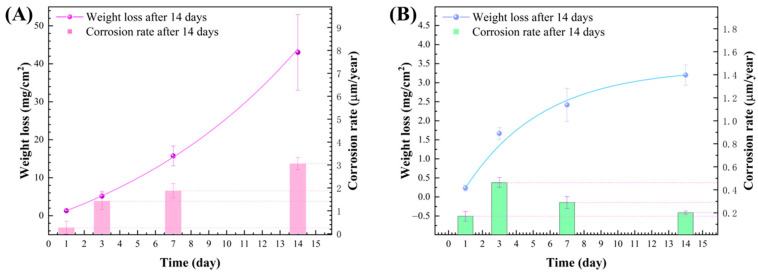
Weight loss and corrosion rate of aluminum alloy coupons during the 14 d incubation in the *D. bizertensis* media (**A**) and sterile culture media (**B**) with different immersion times.

Figure 9 shows the weight loss of aluminum alloy coupons during the 14 d incubation in the *D. bizertensis* media with different immersion times. The ExpDec1 model was used to fit the corrosion weight loss. The corrosion rate of the alloy coupons increased remarkably over immersion times. The weight losses of the aluminum alloy coupons were 1.3 ± 0.2 mg/cm^2^ (0.26 µm/year), 5.1 ± 1.3 mg/cm^2^ (1.42 µm/year), 15.7 ± 2.6 mg/cm^2^ (1.87 µm/year), and 43.1 ± 9.9 mg/cm^2^ (3.05 µm/year) detected at 1 d, 3 d, 7 d and 14 d, respectively. Corrosion rate curves at 1 d, 3 d, 7 d, and 14 d were fitted tangentially in the *D. bizertensis* media, and the corresponding tangent values were −0.41, −0.52, −0.83, and −0.15, respectively. It can be clearly seen that the corrosion accelerated significantly from the 7th day. In the sterile control system, no significant corrosion loss was observed in the aluminum alloy coupons.

### 3.6. Electrochemical Measurements

**Figure 10:**
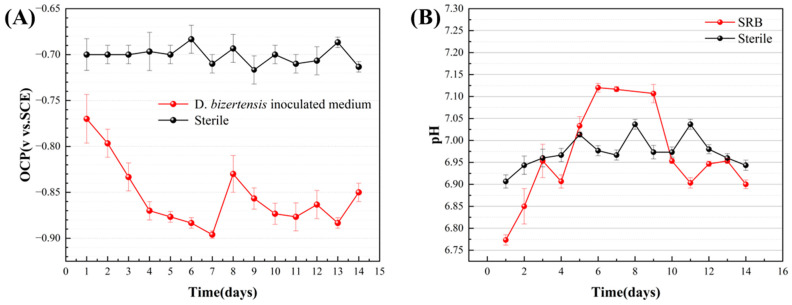
The OCP (**A**) and pH value (**B**) of aluminum alloy coupons during the 14 d incubation in the *D. bizertensis* media with different immersion times.

The OCP variation of aluminum alloy coupons during the 14 d incubation in the *D. bizertensis* media with different immersion times was analyzed (Figure 10). As the immersion time increases, the OCP gradually shifts negatively to −900 mV, implying that the presence of *D. bizertensis* leads to a significant increase in the corrosion activity of aluminum alloy coupons. In the sterile control system, OCP values did not fluctuate significantly.

**Figure 11:**
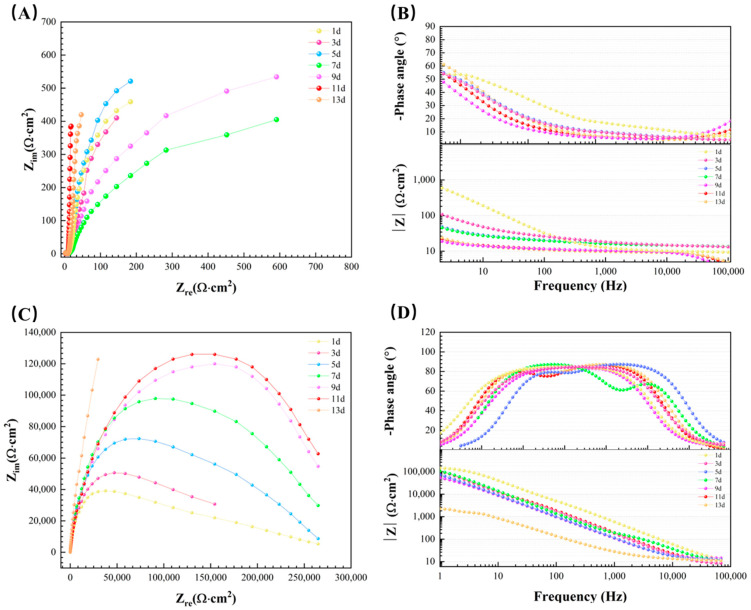
Nyquist (**A**,**C**) and Bode diagrams (**B**,**D**) of aluminum alloy coupons exposed in the *D. bizertensis* (**A**,**B**) and sterile (**C**,**D**) culture media during the 14 d with different immersion times.

The EIS behavior of aluminum alloy coupons during the 14 d incubation in the *D. bizertensis* media with different immersion times was further analyzed (Figure 11). In the Nyquist plots, the radius of the semicircle obviously increased with time in the initial 9d in the *D. bizertensis* medium. For 9–15 days, the radius of the semicircle was basically unchanged. The radius of the semicircle changed little in a sterile medium. The impedance values decreased over immersion time in the Bode plots, indicating much higher corrosion rates. The impedance values changed little in the sterile medium.

**Figure 12:**
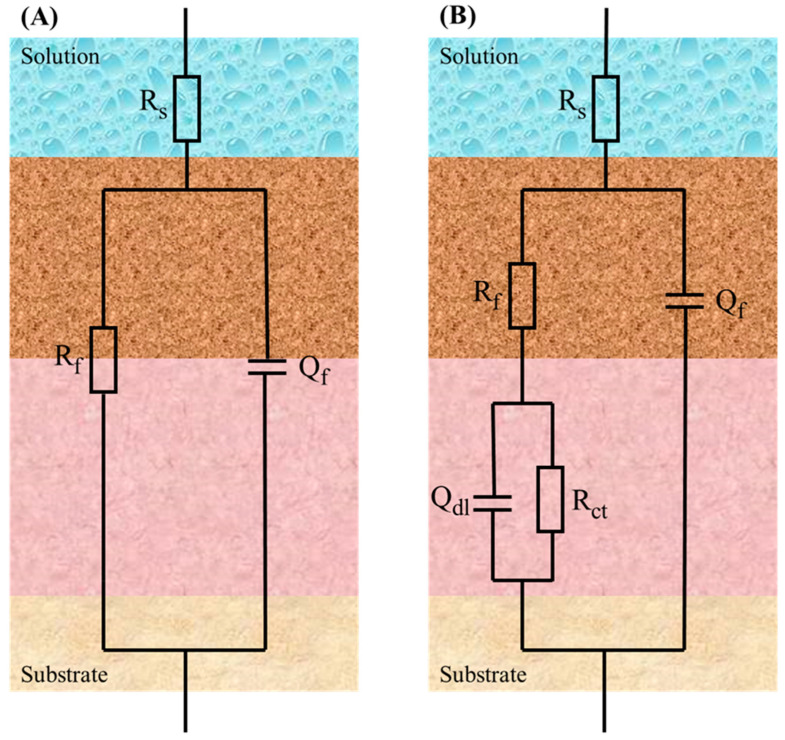
Equivalent electrical circuits used for fitting EIS spectra for aluminum alloy coupons in the *D. bizertensis* media with different immersion times: (**A**): R(QR); (**B**): R(Q(R(QR))).

Figure 12 illustrates the fitting of EIS data using two equivalent circuit models. In the equivalent circuit models, corresponding to R_s_, R_f_, R_ct_, Q_dl_, and Q_f_, the means were the solution, film, charge transfer resistances, the double electric layer, and the capacitances, respectively. The fitted data for the above circuit system are demonstrated in Table 2 and Table 3. By fitting the data, it can be seen that the charge transfer resistance in the sterile control group was significantly higher than that in the *D. bizertensis* media. This is because the dense passivation film formed on the surface of the aluminum alloy prevents corrosion. At the beginning of the immersion, the R_ct_ decreased by an order of magnitude, which is attributed to the accelerated corrosion of the aluminum alloy by the H_2_S produced by the metabolism of SRB.

**Figure 13:**
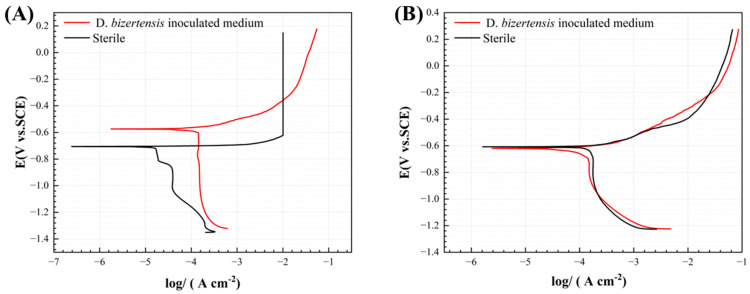
Potentiodynamic polarization curves of aluminum alloy coupons after the 3 d (**A**) and 14 d (**B**) incubations in the abiotic medium and *D. bizertensis* media.

Figure 13 shows the potentiodynamic polarization curves of aluminum alloy coupons after the 3 d and 14 d incubation periods in the *D. bizertensis* media and sterile media, and the corresponding fitting data can be found in Table 4. The corrosion current density of the aluminum alloy coupons was 3.7 µA/cm^2^, 9.8 µA/cm^2^, 51.6 µA/cm^2^, and 69.8 µA/cm^2^ detected at 1 d, 3 d, 7 d and 14 d, respectively. The corrosion current density obviously increased on the 7th day, which was nearly two orders of magnitude higher than that on the 1st day and the 3rd day.

## 4. Discussion

To avoid the detrimental effects caused by the contaminated microbes in aircraft fuel systems, periodic detection and monitoring of the contaminated microbes is an effective way. Based on the recommendations made by the airlines in America, there is a wide range of detection periods for microbial detection, ranging from approximately 1 to 12 months, depending on the number of contaminated microbes in the fuel. An inappropriate testing period leads to increased maintenance costs or heavy security incidents. However, it still lacks a specific standard suitable for microbial corrosion detection in maritime aircraft that face complicated environments. Here, we try to give some suggestions to determine how often the contaminated microbes should be detected. The fundamental logic is like the “wooden barrel effect”—finding the most heavily affected environmental factors as the first step and then performing corrosion tests temporally in the presence of the most corrosive bacterial strains under such harsh conditions. Finally, the time point at which corrosion accelerated significantly was the “key turning point” when microbial detection should be conducted.

### 4.1. Determine the Environmental Factors That Accelerated MIC Most in Aircraft Fuel Systems

Based on the results of the orthogonal experiment, water concentration in the fuel systems significantly impacts the MIC compared with oxygen and temperature (Figure 3). The water content in the fuel can have a significant impact on the growth and metabolism of microorganisms due to the fact that microorganisms require water for all their physiological activities [35]. Despite this, water has both positive and negative effects on MIC in oil–water mixed systems, which depends on the ratio of water and oil. When the water content is below 50%, the activity of microbes is strengthened over the increasing water. However, when the water content is more than 50%, the decreasing fuel concentration also means a reduced nutrient supply [36,37]. Temperature affects the metabolic activity of enzymes in microorganisms, but microorganisms can undergo normal physiological metabolism between 15 °C and 35 °C. Therefore, the temperature in this interval has little effect on MIC. Both increasing oxygen and temperature have a small but negative effect on the MIC of aluminum alloy. This may be explained by the fact that it is easier to form the protective passive film for aluminum alloy against MIC in environments with higher oxygen and temperature.

Unlike the corrosion rate influenced by individual environmental factors, it is more complicated to take all environmental factors into account. According to the analysis of the individual environmental factors, it can be inferred that the most severe MIC should happen in an environment with a water content of 50%, oxygen content of 0%, and temperature of 15 °C. This study clearly shows a different result: MIC was the most serious under conditions with a water content of 50%, oxygen content of 0%, and temperature of 35 °C, according to an orthogonal experiment (Figure 3A). This implies that orthogonal experiments are a very useful way to assess the influence of various environmental factors on MIC in such oil–water systems.

### 4.2. Determination of the Time Node That SRB-Induced Corrosion Is Accelerated Obviously

*D. bizertensis* can degrade fuel oil and obtain nutrient elements such as carbon sources and nitrogen sources to maintain its survival; the acidic metabolites produced by *D. bizertensis* will further lead to the deterioration of fuel oil quality and the corrosion of the aluminum alloy [38,39,40]. The cell counts reached about 10^8^ cells/mL on the 7th day and remained stable around this value (Figure 4). According to the corrosion weight loss and corrosion rate, the corrosion severity of aluminum alloy samples changed greatly from the 7th day (Figure 9). In addition, from the 7th day, the corrosion product layer on the surface of the aluminum alloy specimen was obviously thickened. In summary, the MIC of aluminum alloys in fuel-containing systems increases significantly around 7–9 days. This time node is observed in the presence of most corrosive microbes and in the harshest corrosive environments; hence, it is the “shortest barrel side”. It is recommended to use 7 days as a cycle of fuel detection.

### 4.3. Corrosion Mechanism Analysis of Aluminum Alloy in Fuel–Water Mixed Systems

With the increase in immersion time, both planktonic and sessile *D. bizertensis* cells grew better, as evidenced by the increased planktonic and sessile cell counts (Figure 1) and the denser biofilms in the FM images (Figure 4). The metabolism of *D. bizertensis* converts sulfate to sulfide or H_2_S through a series of reactions. On the one hand, SRB can utilize lactic acid as an electron donor (3a) and sulfate as an electron acceptor (3b) to maintain their metabolism. On the other hand, fuel oil can also be used as a carbon source required for the growth of SRB, which can further accelerate the growth of SRB [41,42].

(3a) $${CH}_{3}{CHOHCOO}^{-}+H_{2}O\to {CH}_{3}{COO}^{-}+{CO}_{2}(g)+4H^{+}+4e^{-}$$

(3b) $${SO}_{4}^{2-}+9H^{+}+8e^{-}\to {HS}^{-}+4H_{2}O$$

In order to prove that the reactions are spontaneous under normal conditions, the equilibrium potential parameters are calculated below [43,44].

(4a) $$E_{e}=-0.0163V-\frac{2.303RT}{F}PH-\frac{RT}{4F}\ln\frac{{CH}_{3}{CHOHCOO}^{-}}{\left({CH}_{3}{COO}^{-}\right)\cdot p_{{CO}_{2}}}\left(vs.SHE\right)$$

(4b) $$E_{e}=0.249V-\frac{2.591RT}{F}PH-\frac{RT}{8F}\ln\frac{{HS}^{-}}{{SO}_{4}^{2-}}\left(vs.SHE\right)\;$$

(5) $$\Delta G=-nFE_{\mathrm{cell}\;}$$

In Equations (4a,b) and (5), R is the universal constant, T is the absolute temperature, F is the Faraday constant, ρ is the partial pressure, and n means a molar quantity. SHE denotes standard hydrogen electrode. At 25 °C, 1 M of solute, 1 bar partial pressure for gasses, and pH 7, for Equation (3a), E_e_ = −430 mV (vs. SHE), and for Equation (3b), E_e_ = −217 mV (vs. SHE). The cell potential of the redox reaction for Equation (3a,b) is E_cell_ = +213 mV. The Gibbs free energy change tends to be negative, as calculated from Equation (4), which means the redox reaction for CH_3_CHOHCOO^−^/CH3COO^−^ and SO_4_^2−^/HS^−^ is thermodynamically favorable.

As a metal with high thermodynamic stability, the Al oxidation reaction is shown below.

(6a) $$Al-3e^{-}\to Al^{3+}$$

(6b) $$E_{e}=-1.66V+\frac{RT}{3F}ln[{Al}^{3+}](vs.SHE)$$

At 25 °C, 1 M solution, 1 bar gasses, and pH 7, E_e_ in Equation (6a) is 737 mV for Al/Al^3+^. This means that the Gibbs free energy change tends to be negative. Thus, the Al corrosion caused by SRB is thermodynamically favorable. Figure 5 shows that the characteristic peaks of Al_2_O_3_ and Al_2_(OH)_3_ were found, but no trace of Al_2_S_3_ was identified because the following reactions may occur.

(7) $${Al}^{3+}+{HS}^{-}+{OH}^{-}\to {Al}_{2}S_{3}+H_{2}O$$

(8) $${Al}_{2}S_{3}+H_{2}O\to Al(OH{)}_{3}+H_{2}S$$

The relative pitting severity (RPS) can be calculated using the formula shown in Equation (9)

(9) $$\mathrm{RPS}\;=\frac{\;\mathrm{maximum}\;\mathrm{pit}\;\mathrm{growth}\;\mathrm{rate}\;}{\;\mathrm{uniform}\;\mathrm{corrosion}\;\mathrm{rate}\;\mathrm{based}\;\mathrm{on}\;\mathrm{specific}\;\mathrm{weight}\;\mathrm{loss}\;}$$

The RPS value is 52.1, 18.3, 32.8, and 29.1, corresponding to 1 d, 3 d, 7 d, and 14 d, respectively. They are all much larger than the unity, indicating that pitting corrosion was dominating in this SRB MIC of aluminum alloy. It has been shown that SRB accelerates the corrosion damage of aluminum alloy by initiating pitting corrosion [45].

## 5. Conclusions

Detection and monitoring of microbial contamination in fuel and fuel systems is an effective strategy to prevent MIC. However, the uncertainty about the detection period of microbial contamination in aviation fuel systems under field conditions poses a significant challenge for frontline managers. The ever-changing environments, with varying oxygen levels, humidity, and temperatures from fuel production to transportation and usage, complicate the detection timeframe. In the present study, we provided a novel solution approach to simplify this issue by integrating orthogonal experiments with corrosion tests. The results suggested microbial contamination should be tested at least every 7 days to prevent corrosion damage occurs. This approach carries practical implications for guiding fuel managers or pilots in implementing measures to minimize microbial contamination in the field when encountering uncertain flying environments. Moreover, this study elucidated the influence of environmental factors on microorganisms, highlighting that water content in fuel oil exerts the most significant impact on the MIC of aluminum alloy. This underscores the importance of controlling water concentration in fuel as the most effective strategy to manage MIC in aircraft fuel systems.

In addition, this study also investigated the corrosion behavior of aluminum alloy when immersed in a marine environment containing *D. bizertensis*. The results of the time gradient experiment over a 14-day immersion period revealed varying effects of SRB on aluminum alloy corrosion and fuel quality at different growth stages. Notably, during the 7–10-day period when the SRB population was most stable, the corrosion rate of the aluminum alloy peaked, accompanied by a significant degradation in fuel quality. Thermodynamic and RPS analyses indicated that the MIC of the aluminum alloy primarily stemmed from acidic substances produced during SRB metabolism under the unique conditions in the present study, which further accelerated corrosion through pitting corrosion amplification. The above results were based on the research on a marine SRB. However, due to the complexity of microorganisms in fuel systems, the synergistic contribution of multiple strains, including bacteria and fungi, to aluminum alloy corrosion in practical working environments still requires in-depth research.

## Figures and Tables

**Table 1:** Nine groups of orthogonal experiments under different environmental conditions.

Group	Water Content	Oxygen Content	Temperature (°C)
1	20%	10%	15
2	20%	20%	35
3	20%	0%	25
4	50%	20%	15
5	50%	10%	25
6	50%	0%	35
7	90%	10%	35
8	90%	0%	15
9	90%	20%	25

**Table 2:** Electrochemical parameters fitted from the electrochemical impedance data of aluminum coupons in the sterile culture media.

Time	*R_S_* (Ω cm^2^)	*Q_f_* × 10^−5^ (Ω^−1^ cm^2^ S^n^)	*R_f_* (Ω cm^2^)	*Q_dl_* × 10^−5^ (Ω^−1^ cm^2^ S^n^)	*R_ct_* (KΩ cm^2^)
1	12.51 ± 0.89	3.89 ± 0.69	73.21 ± 16.08	5.84 ± 1.20	14.98 ± 2.38
3	10.77 ± 0.61	1.49 ± 2.52	33.62 ± 5.58	6.06 ± 1.42	19.66 ± 3.52
5	12.72 ± 0.82	1.86 ± 1.60	50.66 ± 5.10	3.72 ± 2.18	26.82 ± 1.51
7	11.32 ± 1.49	2.99 ± 0.95	59.85 ± 2.91	4.27 ± 0.38	16.92 ± 2.82
9	16.92 ± 0.41	5.58 ± 3.86	57.27 ± 4.10	4.92 ± 1.52	15.87 ± 4.14
11	18.44 ± 2.02	2.67 ± 1.40	22.69 ± 6.10	4.74 ± 1.14	23.12 ± 1.58
13	11.08 ± 0.18	4.09 ± 0.21	26.12 ± 2.92	9.20 ± 1.04	16.69 ± 1.73

**Table 3:** Electrochemical parameters fitted from the electrochemical impedance data of aluminum coupons in the *D. bizertensis* media.

Time	*R_S_* (Ω cm^2^)	*Q_f_* × 10^−5^ (Ω^−1^ cm^2^ S^n^)	*R_f_* (Ω cm^2^)	*Q_dl_* × 10^−5^ (Ω^−1^ cm^2^ S^n^)	*R_ct_* (KΩ cm^2^)
1	8.61 ± 0.25	22.39 ± 3.23	21.38 ± 2.78	3.03 ± 0.41	22.37 ± 1.48
3	8.75 ± 0.54	41.65 ± 3.11	7.62 ± 6.67	18.91 ± 1.23	10.93 ± 0.88
5	10.11 ± 1.33	54.13 ± 3.43	4.05 ± 6.81	14.94 ± 1.74	8.35 ± 1.19
7	11.94 ± 1.93	19.48 ± 2.94	25.02 ± 4.24	11.62 ± 1.43	3.74 ± 0.31
9	9.63 ± 0.97	53.90 ± 2.45	31.24 ± 3.4	10.34 ± 11.88	3.58 ± 0.43
11	14.61 ± 0.19	69.61 ± 0.68	44.93 ± 8.71	32.47 ± 1.73	5.93 ± 0.47
13	19.10 ± 3.97	63.70 ± 6.96	30.89 ± 3.56	28.42 ± 4.56	8.16 ± 1.04

**Table 4:** Electrochemical parameters fitted from the potentiodynamic polarization curves of aluminum coupons in the *D. bizertensis* media.

System	Time	E_corr_/V	I_corr_ × 10^−5^/A/cm^2^	E_pit_/V	∆E_p_/V
Sterile	3	−0.6671 ± 0.04	1.51 ± 0.43	−0.5572 ± 0.12	0.1099 ± 0.07
	14	−0.5922 ± 0.01	4.21 ± 0.96	−0.3981 ± 0.01	0.2150 ± 0.03
SRB	3	−0.7658 ± 0.06	0.91 ± 0.19	−0.4332 ± 0.08	0.4673 ± 0.01
	14	−0.6254 ± 0.01	6.42 ± 3.08	−0.4724 ± 0.03	0.3485 ± 0.24

## Data Availability

The original contributions presented in the study are included in the article, further inquiries can be directed to the corresponding authors.
